# A Sensitive High-Throughput Assay for Evaluating Host-Pathogen Interactions in *Cryptococcus neoformans* Infection

**DOI:** 10.1371/journal.pone.0022773

**Published:** 2011-07-28

**Authors:** Deepa Srikanta, Meng Yang, Matthew Williams, Tamara L. Doering

**Affiliations:** Department of Molecular Microbiology, Washington University School of Medicine, St. Louis, Missouri, United States of America; University of Minnesota, United States of America

## Abstract

**Background:**

*Cryptococcus neoformans* causes serious disease in immunocompromised individuals, leading to over 600,000 deaths per year worldwide. Part of this impact is due to the organism's ability to thwart what should be the mammalian hosts' first line of defense against cryptococcal infection: internalization by macrophages. Even when *C. neoformans* is engulfed by host phagocytes, it can survive and replicate within them rather than being destroyed; this ability is central in cryptococcal virulence. It is therefore critical to elucidate the interactions of this facultative intracellular pathogen with phagocytic cells of its mammalian host.

**Methodology/Principal Findings:**

To accurately assess initial interactions between human phagocytic cells and fungi, we have developed a method using high-throughput microscopy to efficiently distinguish adherent and engulfed cryptococci and quantitate each population. This method offers significant advantages over currently available means of assaying host-fungal cell interactions, and remains statistically robust when implemented in an automated fashion appropriate for screening. It was used to demonstrate the sensitivity of human phagocytes to subtle changes in the cryptococcal capsule, a major virulence factor of this pathogen.

**Conclusions/Significance:**

Our high-throughput method for characterizing interactions between *C. neoformans* and mammalian phagocytic cells offers a powerful tool for elucidating the relationship between these cell types during pathogenesis. This approach will be useful for screens of this organism and has potentially broad applications for investigating host-pathogen interactions.

## Introduction

*Cryptococcus neoformans* is an opportunistic fungal pathogen of mammals, which causes life-threatening illness in severely immunocompromised hosts. Inhalation of the infectious particle results in a primary pulmonary infection that can lead to a fatal meningitis [1]. Cryptococcosis affects close to one million people annually and kills over 600,000 of them, mainly in sub-Saharan Africa [2]. This virulence is mediated by multiple factors, but prominent among them is the ability to form an anti-phagocytic polysaccharide capsule [3].

The first step of cryptococcal infection occurs when a mammalian host inhales the infectious particles, which are of a size that allows them to reach the alveoli. Fungi can then persist and replicate in the alveolar spaces, or they may encounter host macrophages and become internalized [4]–[6]. These infected macrophages may remain in the lungs or leave the pulmonary system, allowing fungal dissemination. Once within macrophages, there are several possible fates for *C. neoformans*. The fungus can exit the macrophage by causing host cell lysis. Alternatively, it can remain sequestered within the host cell, where it can either continue to replicate or potentially exist in a latent form until reactivation in the setting of immune compromise [7]–[9]. The fungus may also be killed by the macrophage, or exit the host cell through an intriguing non-lytic mechanism that may also allow direct transfer between host cells [10]–[13]. Understanding the interactions between mammalian host macrophages and *C. neoformans* is key to explaining successful fungal pathogen dissemination, latency, and host damage [14]–[18].

Host-microbe interactions at the cellular level can be investigated in multiple ways [19]–[22]. We have used microscopy to quantitate the initial interactions between *C. neoformans* and host cells: cell adherence and fungal internalization. Although direct imaging of these events may be possible in some model organisms that have been used to study cryptococcal infection, such as *Caenorhabditis elegans* [20], we have chosen to assay cells in culture to facilitate automation and high-throughput approaches. Multiple systems have been used to study fungal engulfment by phagocytes in culture, ranging from single celled organisms like *Acanthamoeba* and *Dictyostelium* to cell lines derived from *Drosophila*, mouse, and human. While most studies of *C. neoformans* phagocytosis have been performed in murine cell lines, we chose human cell lines as the phagocytic partner in our assay because of the significant human disease caused by this organism.

A variety of methods have been used to quantitate *in vitro* studies of interactions between intracellular pathogens and host cells. Some of these measure total pathogens associated with host cells: for example by exposing host cells to the infecting microbe, washing them, and then assessing associated colony forming units (CFU) [23]; or by using flow cytometry to sort host cells exposed to fluorescent microbes [24], [25]. Although these methods are useful, they generally do not differentiate between adherent and internalized organisms, which are distinct populations in terms of host interactions. One approach to specifically assessing internalized microbes is to add a non-membrane permeant drug to the assay, such that adherent microbes are killed and therefore not viable in CFU assays [26]–[28]. While extremely powerful [29], this method does not allow direct measurement of adherent cells. For directly measuring both adherent and internalized microbes, judicious use of fluorescent staining in conjunction with light microscopy has been most effective [30], [31]; we have applied such an approach below.

Fungal pathogens are an emerging threat for which we have a limited toolbox. These pathogens are evolving rapidly, and severely affect both immunocompromised and immunocompetent individuals [2], [32]–[36]. We have established a new, fast, and accurate method for studying the initial interactions of *C. neoformans* cells with host macrophages. This method offers a powerful approach to understanding cryptococcal biology and has potential application to other pathogens.

## Results

### Assay development

**Figure 1:**
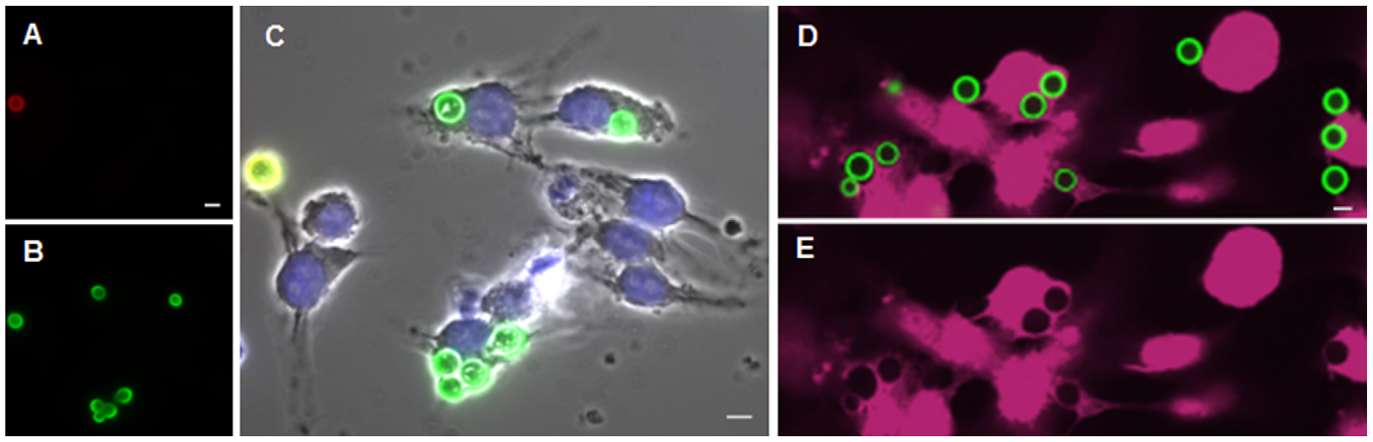
Distinguishing adherent and internalized cryptococci. Panels A–C, J774.16 cells were exposed to fungi labeled with anti-capsular mAb and adherent fungi were labeled with an Alexa Fluor 546-labeled secondary antibody (red). Host cells were then permeabilized, and the samples were restained with an Alexa Fluor 488-labeled secondary antibody to stain all fungi (green). Panel A, red channel; Panel B, green channel; Panel C, merge with DIC and DAPI-stained images. Panels D–E, Confocal images of THP-1 cells were challenged for 1 hr with Lucifer Yellow-stained fungi and stained with CellMask (pink). All fungi appear green in the merged image (D), but only engulfed fungi exclude the cytosolic dye and appear as dark silhouettes in the pink channel (E). Scale bars, 5 µm.

Our goal was to develop a rapid and effective method to differentiate between adherent and engulfed cells after *in vitro* exposure of host cells to *C. neoformans*, and to quantitate each population. We began with a mouse macrophage-like cell line, J774.16, which has been extensively evaluated for interactions with *C. neoformans*. To assess adherence and internalization, we first tested a strategy that has been effective in *C. neoformans* [37]–[39] and other eukaryotic pathogens [40]: performing antibody labeling before and after host cell permeabilization. To do this we exposed host cells to serum-opsonized *C. neoformans* cells, and then stained the samples with an anticapsular monoclonal antibody (3C2 [41], generously provided by T. Kozel) to identify cryptococci that only attach to the host cell surface (Fig. 1A). We next washed the samples, permeabilized the host cells with saponin, and restained the samples using the same antibody tagged with a second fluorophore to label all cryptococci associated with the host cells (Fig. 1B). As shown in the merged image (Fig. 1C; which also shows a DIC image and DAPI-staining of the J774.16 cells), a doubly-labeled adherent yeast (yellow) is clearly distinguishable from the internalized cells that are only labeled with the second fluorophore (green). While this method was clearly effective, and could be scored by automated microscopy (not shown), it requires multiple staining steps and relies on a biological reagent that is organism-specific and not commercially available. For these reasons we considered other methods for identifying fungal cells.

**Figure 2:**
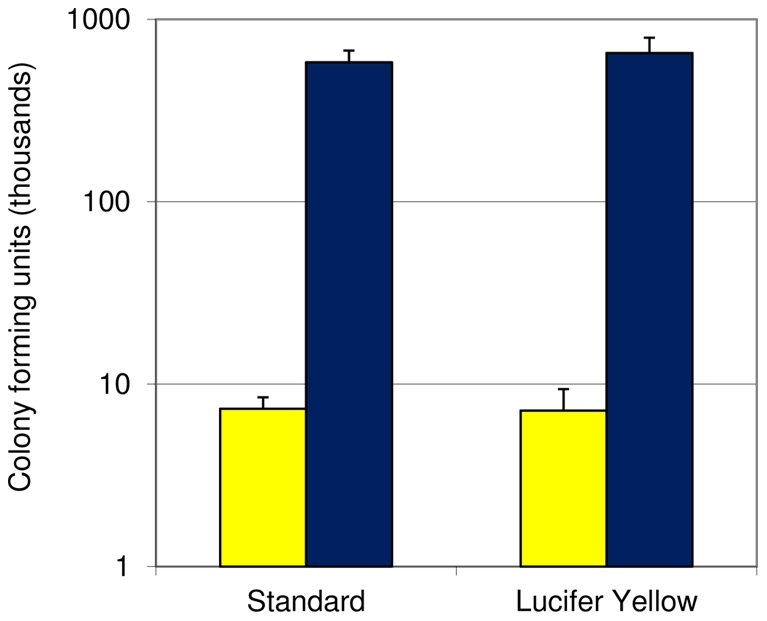
Lucifer Yellow does not alter cryptococcal survival in mouse lung. Mice were infected with fungal cells treated without (standard) or with Lucifer Yellow. Lungs were harvested for CFU counts at 1 hour (yellow bars) or 1 week (blue bars) after infection. Mean and standard deviation of values are shown.

We had previously observed that Lucifer Yellow dye uniformly stains cryptococcal cell walls without affecting cell morphology or subsequent growth in culture (A. Yoneda and T.L. Doering, unpublished observations). This staining is rapid, inexpensive, and commercially available. Before applying this reagent to our studies, however, we needed to be sure that this staining did not adversely affect the fungal cells in terms of their host interactions. To test this, we performed sequential antibody labeling studies (as in Fig. 1A–C) using fungi with or without prior Lucifer Yellow staining. Staining of the fungi before host cell exposure did not alter adherence and uptake as measured by this assay (Table S1). We next tested Lucifer Yellow-stained cryptococci in a mouse model of infection (see Methods). We found that the stained fungal cells were able to proliferate in the mouse lungs to the same degree as the untreated fungal cells (Fig. 2; p value = 0.37), suggesting that the dye caused no significant alteration in their host interactions or growth.

Based on our results in tissue culture and in mice, we used Lucifer Yellow-stained cryptococci to assay interactions with THP-1, a human monocytic cell line [42], [43] that has been used to study host cell interactions with other pathogenic microbes [44]–[47]. We exposed THP-1 derived macrophages to Lucifer Yellow-stained fungi, washed them with PBS to remove non-adherent cells, and then stained the host cytosol with CellMask. As shown by confocal fluorescent imaging (Fig. 1D), both cell types are easily visualized, and the displacement of the cytosolic dye by internalized fungi (Fig. 1E) allows those that have been engulfed (seen as silhouettes in the host cytosol) to be readily distinguished from those that are adherent to the host cell surface. We also analyzed assay samples in parallel using this method or the method described above that is based on antibody staining before and after host cell permeabilization. The results from the two methods were indistinguishable (not shown).

**Figure 3:**
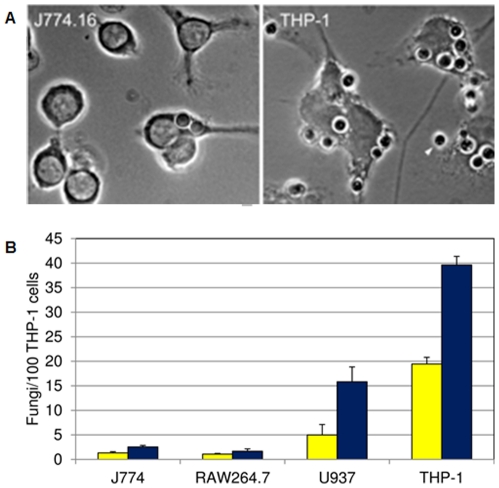
Host lines differ in phagocytosis of *C. neoformans*. Panel A, bright-field microscopy of J774.16 and THP-1 cells challenged with fungi. All fungal cells shown were internalized except one (yellow arrowhead). Scale bar, 5 µm. Panel B, adherence (yellow bars) and uptake (blue bars) values for the indicated macrophage-like cell lines. Mean and standard deviation values are shown.

While developing our assay, we observed that fungi interacted more extensively with THP-1 cells than with J774.16 (Fig. 3A). To quantitate this result and explore additional host lines, we compared the interactions between *C. neoformans* and J774.16, THP-1, RAW 264.7 (a murine macrophage-like cell line [48]), and U937 (a human monocytic cell line [49]). All of these cell lines share characteristics important for our assay: they display receptors involved in the complement pathway (known to be important in cryptococcal infection [50]–[53]), and they have phagocytic capabilities. In a direct comparison we found that THP-1 cells were most active in our assay (Fig. 3B), so we used this human cell line for all subsequent studies.

### Automated imaging and assay kinetics

**Figure 4:**
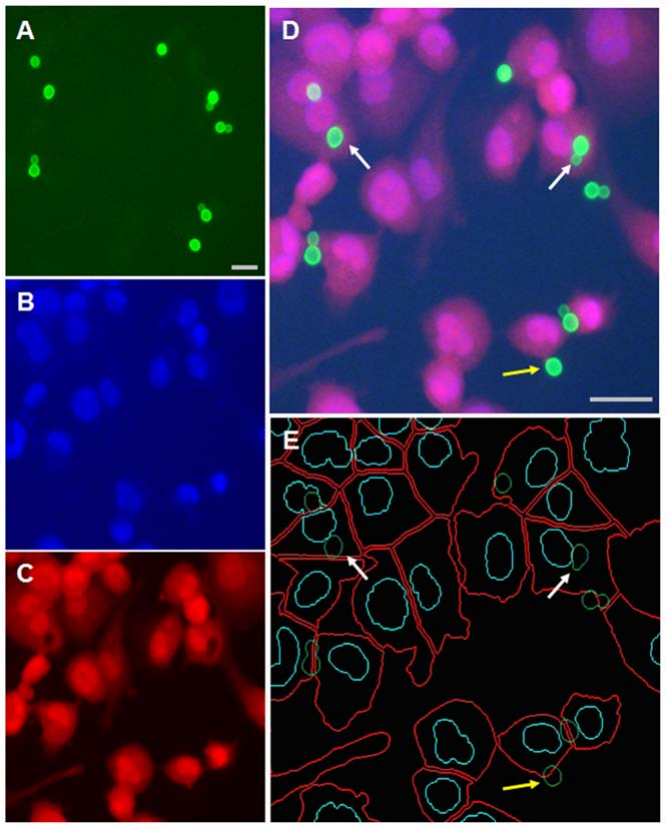
Automated fluorescent microscopy and segmentation analysis. Uptake of Lucifer yellow-stained fungal cells by THP-1 cells was performed as described in Methods. Panels A–C, cryptococcal cells imaged by Lucifer Yellow (A), host nuclei imaged with DAPI (B), and host cytosol imaged by CellMask (C). Panel D, fused image of Panels A–C. Panel E, the image in panel D automatically annotated to indicate boundaries of host cells (red), host cell nuclei (turquoise), and fungi (green). Fungal cells overlapping the cell body by less than 50% are considered adherent (yellow arrow) and those overlapping by ≥50% are considered engulfed (white arrow). The fungal cell at upper left appears pale because it overlies the host nucleus. Scale bars, 10 µm.

Our studies up to this point were analyzed manually, with visualization by conventional fluorescence microscopy. While this method yielded clear and reproducible results, we wished to automate our assays to reduce the time required for analysis. To do this we took advantage of automated high-content imaging, after first scaling our assay up to 96-well microtiter plate format. As in the earlier studies, we challenged host cells with Lucifer Yellow-stained cryptococci, then fixed and stained the samples with DAPI and CellMask. We next imaged the wells with a GE INCell Analyzer, using individual channels to visualize Lucifer Yellow-stained fungi (Fig. 4A), DAPI-stained host nuclei (Fig. 4B), and CellMask-stained host cytosol (Fig. 4C). The relationship between these stains is clearly seen in a merged image of a representative assay (Fig. 4D). Finally, we used an automated developer to identify each stained entity (Fig. 4E), and to classify each fungal cell in terms of its relationship to the host cells. We defined adherent cells as those where signal overlap with a host cell was greater than 1% and less than 50%, and engulfed cells as those where the overlap was equal to or greater than 50%, recording all results on an individual cell basis to allow flexible analysis. We recognize that this classification may not always be precise because of cell positions in the imaging field, but parallel manual inspection (including examination of multiple focal planes) yielded results that were statistically indistinguishable (Table S2). This suggests that we have developed a robust and biologically meaningful assay.

**Figure 5:**
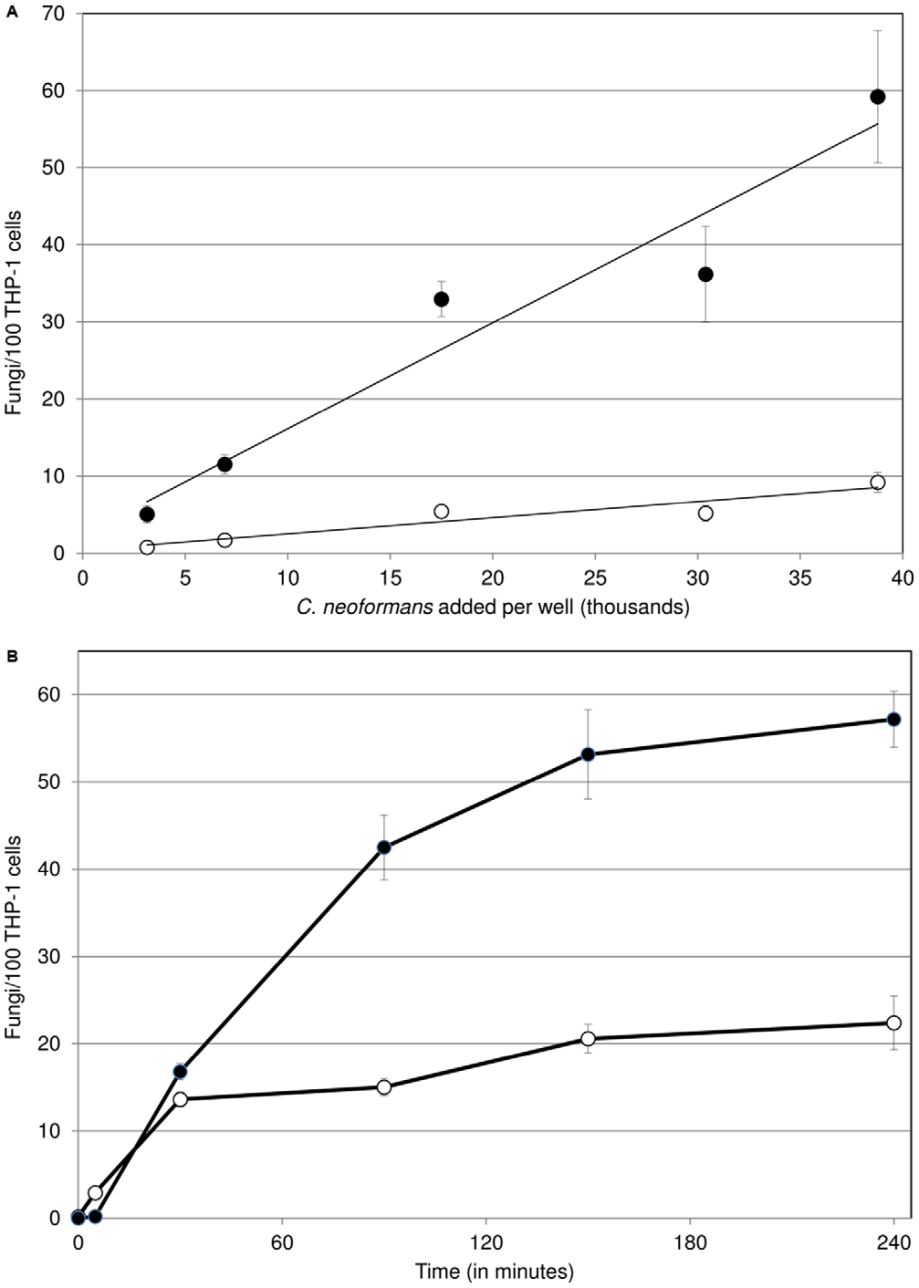
Effects of cell ratio and exposure time on adherence and uptake. Indices of adherence (open circle) and uptake (closed circle) vary with the number of fungal cells added per well (A) and with time (B). Mean and standard deviation are plotted (some error bars are too small to see).

Having established a reliable assay with automated analysis, we used it to characterize various experimental parameters. Among these we noted a linear relationship between the number of cryptococcal cells added to each host well and their adherence or uptake by the phagocytes, up to a ratio of at least 1∶1 (Fig. 5A). We also performed a time course study to examine the kinetics of interactions with the host cells. We found that adherence begins to level off close to 30 minutes after exposure, while uptake does not begin to plateau until significantly later (Fig. 5B; see Discussion).

### Biological and statistical assay validation

**Figure 6:**
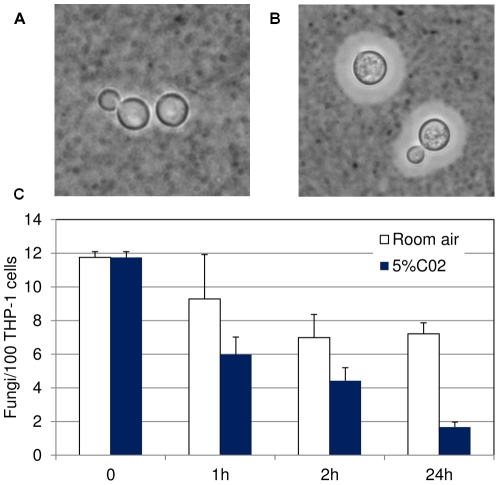
Cryptococcal host interactions are highly sensitive to capsule induction. Upper panels, India ink staining of fungal cells at 24 h without (A) or with (B) capsule induction. Panel C, standard assay performed with cells grown for the indicated times in the conditions shown. Mean and standard deviation are plotted for indices of adherence. All samples were grown at 37°C in either room air (white bars) or 5% CO_2_ (blue bars).

We next wanted to apply our assay to a feature of *C. neoformans* with known biological relevance to phagocyte interactions. The cryptococcal capsule, mentioned above as a major virulence factor of this pathogen, is known to be antiphagocytic [53]–[56]. Capsule size increases during growth in various inducing media, including the ‘host-like’ conditions of growth in mammalian tissue culture medium at 37°C in a 5% CO_2_ atmosphere. Enlarged capsule can be readily observed as a halo surrounding the cell wall upon negative staining with India ink (Fig. 6B), and is typically visible by such staining after 4–8 hours in inducing conditions (not shown). We grew cells in parallel under these inducing conditions or in the same medium at 37°C but in room air (which does not yield appreciable capsule, Fig. 6A), and removed samples at various time points for testing in our assay. We saw reduced adherence as early as 1 hour after the start of capsule induction (Fig. 6C), a time point at which changes in capsule size are not visible by India ink staining (not shown). Both adherence and uptake were almost completely repressed in capsule induced samples by 24-hours (Fig. 6C and data not shown), a time point at which uptake and adherence of uninduced samples is still robust.

**Figure 7:**
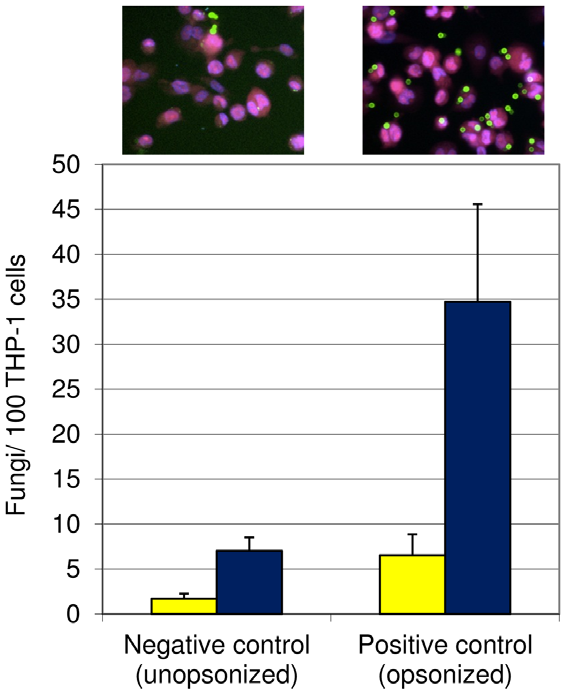
Positive and negative controls used for statistical analysis of assay power. Standard assays were performed using the controls shown above. Mean and standard deviation are plotted for adherence (yellow bars) and uptake (blue bars). Representative images for each control are shown above the bars. Scale bar, 5 µm.

The experiments described above, including the capsule induction study, demonstrated that our automated assay is rapid and sensitive to cellular features of biological significance, suggesting it will be appropriate for large-scale screening projects. For such studies, however, it is necessary to statistically characterize and validate the assay. To do this, we assayed sets of 96-well plates in triplicate using unopsonized and opsonized cryptococcal cells as negative and positive control conditions, respectively (Fig. 7). We then evaluated our assay with two different statistical measures, Z factor [57] and SSMD (strictly standardized mean difference [58]–[60]). Z factor compares the variation in average assay values for positive and negative controls to the difference between those values, and is best applied to studies in which a normal distribution of data is expected. We routinely observed Z factor scores between 0.5 and 1, indicating an excellent screening assay, appropriate for high-throughput studies. SSMD relates the difference between the mean values of two control populations to the standard deviation of the difference between them [58], and is more robust to variation in data ranges (outliers) and to data variability. Our calculated SSMD scores were typically above 3 (Table S3), which, like the results for Z-factor, demonstrate that the assay is appropriate for high-throughput screening.

## Discussion

Central features of cryptococcal pathogenesis, including dissemination, latency, and host cell damage, depend upon the interactions between *Cryptococcus neoformans* cells and mammalian phagocytes. Screening approaches are potentially powerful for elucidating these processes. However, current ways to assess fungal-host cell interactions, such as protocols that rely on low-throughput imaging techniques and limited reagents [37], [61], are not ideal for screens. To enable efficient screening of interactions between *C. neoformans* and host phagocytes, we have applied high-content automated microscopy and analysis. This allows us to assess the initial interactions of *Cryptococcus neoformans* with mammalian host macrophages in a high-throughput manner.

A key parameter in this work was the choice of host cell type, so we investigated the uptake and adherence indices of four macrophage/macrophage-like cell lines, ultimately choosing the human THP-1 cell line for our assay. Although most studies of *C. neoformans* have used mouse lines such as J774.16, we chose to work within the human system, using both human serum and a human cell line. THP-1 cells may be readily differentiated into macrophage-like cells in culture and demonstrate a high phagocytic capability in our assay, even without activation by antibodies or LPS (which have been frequently used for cryptococcal uptake studies). Importantly, these cells display Fc and C3 receptors, which play roles in pathogen recognition; the latter are particularly important for recognition of the cryptococcal capsule [50]. We have validated the biological sensitivity of THP-1 cells to *C. neoformans* by our demonstration that they can detect alterations in capsule within less than two hours of capsule induction by ‘host-like’ conditions.

Our protocol readily distinguishes between adherent and engulfed cryptococci in an efficient and cost-effective manner. It requires fewer manipulations than antibody-based protocols, reducing cell loss as well as effort and reagents. The method directly images the particles of interest, in contrast to studies that use antibodies or quenching [23] to calculate pathogen distribution based on differences in signal. Importantly, this assay may also be readily adapted to multiple cell types by using widely available stains to label the host nuclei and cytosol as well as the cryptococcal cells. The ability to simultaneously track adherent and internalized cells has allowed us to observe differences in the kinetics of appearance of these populations (Fig. 5). These results suggest that adherence precedes internalization, as might be expected, and further that these processes may be saturable. The ability to differentiate between bound and internalized populations, which is not currently possible with other high-throughput assays, allows us to study these distinct steps independently.

The largely automated protocol we have developed has multiple technical advantages. Up to eighty samples plus controls can be screened simultaneously in 96-well format, and higher density formats offer promising preliminary results (not shown). Our statistical analysis reveals consistent results across and within multiple plates, and the assay is appropriate for high-throughput screening based on two different statistical parameters (Z factor and SSMD). We are able to minimize the subjectivity that is associated with microscopy based assays by using automated imaging, and the high-content images generated in this way are rich in information which can be mined for additional phenotypic details. We have validated the method through the use of different cell types, both host (Fig. 2) and pathogen (not shown), and the data quality matches that of the “gold standard” method of using manual inspection of antibody-labeled samples (Table S2). Finally, our automated collection of data on individual host and fungal cells offers great flexibility in terms of analysis.

*Cryptococcus neoformans* is a successful fungal pathogen. Much of this is due to its ability to produce capsule polysaccharides, which impede host defenses including phagocytic cell deployment and phagocytosis. Multiple aspects of the latter process may be studied using variations of our assay. For example, we have an excellent vantage point for the initial stages of host cell entry, which also avoids the potential complication of fungal growth. We can change that view by altering the time from our current one hour exposure, potentially using shorter co-incubations to study the earliest fungal-host cell interactions. Similarly, we can use later time points to assess downstream events, perhaps including intracellular fungal replication [36] or fungal cell expulsion [10], [11]. We can also examine perturbation of the fungal-host interactions by assessing the effects on this assay of introducing cryptococcal mutants, down-regulating host genes, or adding various drugs. Together, this assay and these variations will increase our understanding of key events in cryptococcal pathogenesis.

## Materials and Methods

### Ethics Statement

All animal protocols were conducted following the guidelines found in the Guide for the Care and Use of Laboratory Animals of the National Institutes of Health and were approved by Washington University School of Medicine DCM (#20080269). All efforts were made to minimize animal suffering.

### Yeast cell growth and conditions

*C. neoformans* strain H99 (cultured from the ATCC *C. neoformans* deletion collection, #208821) was grown in YPD broth (1% Yeast extract, 2% peptone and 2% dextrose) and maintained at 30°C on YPD plates containing 2% agar. To induce capsule formation, cultures were grown overnight from single colonies in YPD and the cells were harvested and washed in DMEM (Sigma). The cell density was measured using a hemocytometer and the culture was diluted in DMEM to 1.2×10^7^/ml in a tissue culture flask. The flasks were incubated at 37°C in a 5% CO_2_ incubator to induce *C. neoformans* capsule formation or in room air for control cultures. Capsule formation was assessed by India ink staining as in [62].

### Mammalian cells and growth conditions

All mammalian cell lines were grown and maintained at 37°C under 5% CO_2._ Human monocytic cell lines THP-1 and U937 were obtained from J. Atkinson and J. Vogel, respectively, and cultured in RPMI-1640 (Invitrogen) supplemented with 10% heat-inactivated FBS (Gibco), 100 µg/ml penicillin and 100 U/ml streptomycin (Pen/Strep; Gibco), 1 mM sodium pyruvate (Cellgro), and 48 µM β-mercaptoethanol (Fisher Scientific). Murine macrophage-like cell lines J774.16 and RAW264.7 were obtained from the ATCC and D. Sibley, respectively, and cultured in DMEM (Sigma) supplemented with 10% heat-activated FBS and Pen/Strep. Monocytic cells were passaged every two days to maintain cell density between 1.5×10^5^/ml and 9×10^5^/ml. Macrophage-like cells were passaged every three days.

### Macrophage uptake assessed by manual counts

For manual uptake assays, freshly harvested J774.16 cells were washed twice in DMEM, adjusted to 8.3×10^5^/ml in DMEM, and 300 µl aliquots of the suspension were seeded onto 12 mm silanized coverslips (Ingen Lab) in 24-well tissue culture plates (TPP) and incubated at 37°C/5% CO_2_ for 24 hours. In parallel, overnight cultures of *C. neoformans* in YPD were washed twice in PBS and adjusted to 4.2×10^6^/ml in PBS. 240 µl of the yeast cell suspension were opsonized and labeled by mixing with 160 µl human serum (from healthy volunteers, following a protocol approved by the Washington University School of Medicine IRB), followed by the addition of 2 µg anti-cryptococcal capsule antibody 3C2 (from T. Kozel). After a 30 min incubation (37°C, room air, with rotation) the cells were washed three times in PBS and re-suspended at 3.3×10^6^ cells/ml in DMEM (Fig. 1).

To perform the assays, prepared J774.16 macrophages were washed gently once with DMEM to remove non-adherent cells, and 10^6^ prepared fungal cells were added to each well. The plates were incubated at 37°C/5% CO_2_ for 4 hours, washed three times with PBS to remove excess fungal cells, fixed for 10 min on ice with 4% formaldehyde in PBS, and washed twice with PBS. To stain fungal cells adhering to the surface of the macrophages, 300 µl Alexa Fluor 546-conjugated goat anti-mouse IgG antibody (Invitrogen) in PBS (25 µg/ml final concentration) was added to each well and the plates were incubated at 37°C/5% CO_2_ for 30 min and then washed three times with PBS. Macrophages were next permeabilized by incubating for 30 min at 25°C with 300 µl 0.1% saponin in PBS, washed with PBS, and stained as before but using 10 µg/ml Alexa Fluor 488-conjugated goat anti-mouse IgG antibody (Invitrogen). Finally, 300 µl of 1 µg/ml DAPI in PBS was added to each well to stain macrophage nuclei and the plates were incubated for 15 min at 25°C and washed four times with PBS.

For visualization, 10-µl drops of Prolong Gold (Invitrogen) were placed onto microscope slides (VWR) and the coverslip from each well of the tissue culture plate was inverted onto one drop, incubated at 25°C overnight in the dark, and observed by fluorescence microscopy to determine the index of adherence (adherent fungi per 100 macrophages) and the phagocytic index (ingested fungi per 100 macrophages). Triplicate counts were performed on each well, samples were tested in triplicate, and each experiment was repeated at least three times.

### Automated assessment of macrophage uptake and adherence

For automated assays using monocytic cell lines, THP-1 or U937 cells were harvested and then induced to differentiate by resuspension at 3.4×10^5^/ml in growth medium supplemented with 0.2 µg/ml phorbol 12-myristate 13-acetate (PMA) (Sigma). Flat-bottomed polystyrene 96-well microtiter plates (Costar) were then seeded with 3.4×10^4^ cells per well, incubated at 37°C/5% CO_2_ for 48 hrs, washed three times with RPMI-1640 to remove unattached cells, and incubated for an additional 24 hours in RPMI-1640 containing Pen/Strep and 0.2 µg/ml PMA. (It has recently been suggested that growth with and without PMA be extended to 3 and 5 days, respectively, to generate macrophage-like cells which more closely resemble monocyte-derived macrophages [63]. We find the absolute difference between assay results using cells differentiated by either method is below 4% (Table S4).) For automated assays using macrophage cell lines, RAW264.7 or J774.16 cells were harvested and washed three times in DMEM, re-suspended in DMEM supplemented with Pen/Strep and incubated at 37°C/5% CO_2_ for 24 hours.

In parallel to host cell preparation, 6×10^6^ *C. neoformans* cells grown as above were dispensed into each well of a 96-well microtiter plate. Cells were washed twice in PBS and once in MacIlvaine's buffer, pH 6.0, and then labeled by the addition of Lucifer yellow (Sigma) in MacIlvaine's buffer to each well (100 µg/ml final concentration) and incubation for 30 min at 25°C with shaking on an orbital shaker (BELLCO). The labeled cells were then washed with and resuspended in 1 ml PBS in a 1.5 ml microcentrifuge tube. For opsonization the cells were mixed with one half volume of human serum and incubated at 37°C for 30 min with orbital shaking. Finally, the opsonized fungal cells were washed three times with PBS and resuspended at 1.7×10^6^/ml in RPMI-1640 or DMEM. All uptake experiments with this protocol were opsonized using only human serum.

To perform the assays, the prepared host cells were washed once with RPMI-1640 or DMEM depending on cell type, the medium was aspirated, and 100 µl of the labeled and opsonized fungal cell suspension was added to each well. The co-inoculated plates were incubated at 37°C/5% CO_2_ for 1 hour and washed four times with PBS using an ELx405 Select CW plate washer (BioTek; used for all washes in this paragraph). Cells were fixed as above, washed twice with PBS, permeabilized for 20 min at 25°C with 0.1% saponin in PBS, and washed again with PBS. The macrophage nuclei and cytoplasm were stained for 15 min at 25°C with PBS containing 2 µg/ml DAPI and 250 ng/ml CellMask Deep Red (both from Sigma), washed twice with PBS, and stored in PBS containing 10 mM sodium azide at 4°C in the dark. Plates were imaged using an IN Cell analyzer (GE Healthcare) scanning on channels of wavelengths 360/460, 475/535, and 620/460 (to detect DAPI, Lucifer Yellow, and CellMask, respectively). Images were analyzed using the IN Cell Developer Toolbox (GE Healthcare). Each sample was replicated in multiple wells within one 96-well microtiter plate and/or one well in the same position located on 3 separate 96-well plates. 15–20 counts were performed on each well, and all experiments were repeated at least twice.

### Mouse infection

*C. neoformans* from overnight cultures in YPD were washed twice in PBS and the pellet resuspended in MacIlvaine's Buffer pH 6.0 at a final cell density of 1.3×10^8^/ml. Cells were mixed with 100 µg/ml Lucifer yellow (final concentration) or an equal volume of sterile water (for controls), incubated at 25°C with shaking for 30 minutes, washed twice with PBS, and resuspended in PBS to a final cell density of 2.5×10^5^/ml. For each cell population to be tested, eight female C57BL/6J mice, aged 4–6 weeks, were anesthetized with 150 µl ddH_2_O containing 2 mg/ml xylazine (VEDCO) and 10 mg/ml of ketaset (Fort Dodge Animal Health) by intraperitoneal injection and intranasally inoculated with 50 µl of the fungal suspension (1.25×10^5^ cells). Mice were sacrificed at one hour (3 animals) or one week (5 animals) post infection, and the lungs were harvested and homogenized in 5 ml PBS. Serial dilutions of 50 µl aliquots of the lung homogenate were spotted onto YPD plates, incubated at 30°C overnight, and used to calculate colony forming units (CFU) per animal. In this time period, mice infected by this protocol exhibit no symptoms of illness.

## Supporting Information

Table S1 Cryptococcal adherence and uptake of cells without or with prior staining with Lucifer Yellow. Adherence and uptake were assessed by antibody staining as described in the Methods.
(XLS)

Table S2 Comparison of automated and manual counts on control assays using Lucifer Yellow-labeled fungi and THP-1 cells.
(XLS)

Table S3 Derivation of SSMD values for control studies.
(XLS)

Table S4 Comparison of PMA-differentiation protocols for THP-1 cells.
(XLS)

## References

[1] Chayakulkeeree M, Perfect JR (2006). Cryptococcosis.. Infect Dis Clin North Am.

[2] Park BJ, Wannemuehler KA, Marston BJ, Govender N, Pappas PG (2009). Estimation of the current global burden of cryptococcal meningitis among persons living with HIV/AIDS.. AIDS.

[3] Doering TL (2009). How sweet it is! Cell wall biogenesis and polysaccharide capsule formation in Cryptococcus neoformans.. Annu Rev Microbiol.

[4] Eisenman HC, Casadevall A, McClelland EE (2007). New insights on the pathogenesis of invasive Cryptococcus neoformans infection.. Curr Infect Dis Rep.

[5] Bulmer GS, Sans MD (1967). Cryptococcus neoformans. II. Phagocytosis by human leukocytes.. J Bacteriol.

[6] Diamond RD, Bennett JE (1973). Growth of Cryptococcus neoformans within human macrophages in vitro.. Infect Immun.

[7] Levitz SM, Nong SH, Seetoo KF, Harrison TS, Speizer RA (1999). Cryptococcus neoformans resides in an acidic phagolysosome of human macrophages.. Infect Immun.

[8] Tucker SC, Casadevall A (2002). Replication of Cryptococcus neoformans in macrophages is accompanied by phagosomal permeabilization and accumulation of vesicles containing polysaccharide in the cytoplasm.. Proc Natl Acad Sci U S A.

[9] Garcia-Hermoso D, Janbon G, Dromer F (1999). Epidemiological evidence for dormant Cryptococcus neoformans infection.. J Clin Microbiol.

[10] Ma H, Croudace JE, Lammas DA, May RC (2006). Expulsion of live pathogenic yeast by macrophages.. Curr Biol.

[11] Alvarez M, Casadevall A (2006). Phagosome extrusion and host-cell survival after Cryptococcus neoformans phagocytosis by macrophages.. Curr Biol.

[12] Ma H, Croudace JE, Lammas DA, May RC (2007). Direct cell-to-cell spread of a pathogenic yeast.. BMC Immunol.

[13] Alvarez M, Casadevall A (2007). Cell-to-cell spread and massive vacuole formation after Cryptococcus neoformans infection of murine macrophages.. BMC Immunol.

[14] Goldman DL, Lee SC, Mednick AJ, Montella L, Casadevall A (2000). Persistent Cryptococcus neoformans pulmonary infection in the rat is associated with intracellular parasitism, decreased inducible nitric oxide synthase expression, and altered antibody responsiveness to cryptococcal polysaccharide.. Infect Immun.

[15] Del Poeta M (2004). Role of phagocytosis in the virulence of Cryptococcus neoformans.. Eukaryot Cell.

[16] Voelz K, Lammas DA, May RC (2009). Cytokine signaling regulates the outcome of intracellular macrophage parasitism by Cryptococcus neoformans.. Infect Immun.

[17] Alvarez M, Burn T, Luo Y, Pirofski LA, Casadevall A (2009). The outcome of Cryptococcus neoformans intracellular pathogenesis in human monocytes.. BMC Microbiol.

[18] Oliveira DL, Freire-de-Lima CG, Nosanchuk JD, Casadevall A, Rodrigues ML (2010). Extracellular vesicles from Cryptococcus neoformans modulate macrophage functions.. Infect Immun.

[19] Cummings CA, Relman DA (2000). Using DNA microarrays to study host-microbe interactions.. Emerg Infect Dis.

[20] Mylonakis E, Ausubel FM, Perfect JR, Heitman J, Calderwood SB (2002). Killing of Caenorhabditis elegans by Cryptococcus neoformans as a model of yeast pathogenesis.. Proc Natl Acad Sci U S A.

[21] Kurz CL, Ewbank JJ (2000). Caenorhabditis elegans for the study of host-pathogen interactions.. Trends Microbiol.

[22] Fan W, Kraus PR, Boily MJ, Heitman J (2005). Cryptococcus neoformans gene expression during murine macrophage infection.. Eukaryot Cell.

[23] Walenkamp AM, Scharringa J, Schramel FM, Coenjaerts FE, Hoepelman IM (2000). Quantitative analysis of phagocytosis of Cryptococcus neoformans by adherent phagocytic cells by fluorescence multi-well plate reader.. J Microbiol Methods.

[24] Chaka W, Scharringa J, Verheul AF, Verhoef J, Van Strijp AG (1995). Quantitative analysis of phagocytosis and killing of Cryptococcus neoformans by human peripheral blood mononuclear cells by flow cytometry.. Clin Diagn Lab Immunol.

[25] Voelz K, Johnston SA, Rutherford JC, May RC (2010). Automated analysis of cryptococcal macrophage parasitism using GFP-tagged cryptococci.. PLoS One.

[26] Falkow S, Small P, Isberg R, Hayes SF, Corwin D (1987). A molecular strategy for the study of bacterial invasion.. Rev Infect Dis.

[27] Zhang H, Gomez-Garcia MR, Brown MR, Kornberg A (2005). Inorganic polyphosphate in Dictyostelium discoideum: influence on development, sporulation, and predation.. Proc Natl Acad Sci U S A.

[28] Elsinghorst EA (1994). Measurement of invasion by gentamicin resistance.. Methods Enzymol.

[29] Darfeuille-Michaud A, Boudeau J, Bulois P, Neut C, Glasser AL (2004). High prevalence of adherent-invasive Escherichia coli associated with ileal mucosa in Crohn's disease.. Gastroenterology.

[30] Boleti H, Ojcius DM, Dautry-Varsat A (2000). Fluorescent labelling of intracellular bacteria in living host cells.. J Microbiol Methods.

[31] Sveum RJ, Chused TM, Frank MM, Brown EJ (1986). A quantitative fluorescent method for measurement of bacterial adherence and phagocytosis.. J Immunol Methods.

[32] Dromer F, Ronin O, Dupont B (1992). Isolation of Cryptococcus neoformans var. gattii from an Asian patient in France: evidence for dormant infection in healthy subjects.. J Med Vet Mycol.

[33] Singh N, Dromer F, Perfect JR, Lortholary O (2008). Cryptococcosis in solid organ transplant recipients: current state of the science.. Clin Infect Dis.

[34] Bartlett KH, Kidd SE, Kronstad JW (2008). The emergence of Cryptococcus gattii in British Columbia and the Pacific Northwest.. Curr Infect Dis Rep.

[35] Byrnes EJ, Li W, Lewit Y, Ma H, Voelz K (2010). Emergence and pathogenicity of highly virulent Cryptococcus gattii genotypes in the northwest United States.. PLoS Pathog.

[36] Ma H, Hagen F, Stekel DJ, Johnston SA, Sionov E (2009). The fatal fungal outbreak on Vancouver Island is characterized by enhanced intracellular parasitism driven by mitochondrial regulation.. Proc Natl Acad Sci U S A.

[37] Feldmesser M, Rivera J, Kress Y, Kozel TR, Casadevall A (2000). Antibody interactions with the capsule of Cryptococcus neoformans.. Infect Immun.

[38] Kozel TR (1996). Activation of the complement system by pathogenic fungi.. Clin Microbiol Rev.

[39] Kwon-Chung KJ, Kozel TR, Edman JC, Polacheck I, Ellis D (1992). Recent advances in biology and immunology of Cryptococcus neoformans.. J Med Vet Mycol.

[40] Carruthers VB, Giddings OK, Sibley LD (1999). Secretion of micronemal proteins is associated with toxoplasma invasion of host cells.. Cell Microbiol.

[41] MacGill TC, MacGill RS, Casadevall A, Kozel TR (2000). Biological correlates of capsular (quellung) reactions of Cryptococcus neoformans.. J Immunol.

[42] Tsuchiya S, Yamabe M, Yamaguchi Y, Kobayashi Y, Konno T (1980). Establishment and characterization of a human acute monocytic leukemia cell line (THP-1).. Int J Cancer.

[43] Auwerx J (1991). The human leukemia cell line, THP-1: a multifacetted model for the study of monocyte-macrophage differentiation.. Experientia.

[44] Cossart P, Bierne H (2001). The use of host cell machinery in the pathogenesis of Listeria monocytogenes.. Curr Opin Immunol.

[45] Rogers PD, Thornton J, Barker KS, McDaniel DO, Sacks GS (2003). Pneumolysin-dependent and -independent gene expression identified by cDNA microarray analysis of THP-1 human mononuclear cells stimulated by Streptococcus pneumoniae.. Infect Immun.

[46] Kohler S, Foulongne V, Ouahrani-Bettache S, Bourg G, Teyssier J (2002). The analysis of the intramacrophagic virulome of Brucella suis deciphers the environment encountered by the pathogen inside the macrophage host cell.. Proc Natl Acad Sci U S A.

[47] Hickman-Davis JM, Fang FC, Nathan C, Shepherd VL, Voelker DR (2001). Lung surfactant and reactive oxygen-nitrogen species: antimicrobial activity and host-pathogen interactions.. Am J Physiol Lung Cell Mol Physiol.

[48] Ralph P, Nakoinz I (1977). Antibody-dependent killing of erythrocyte and tumor targets by macrophage-related cell lines: enhancement by PPD and LPS.. J Immunol.

[49] Nilsson K, Sundstrom C (1974). Establishment and characteristics of two unique cell lines from patients with lymphosarcoma.. Int J Cancer.

[50] Kozel TR, Wilson MA, Murphy JW (1991). Early events in initiation of alternative complement pathway activation by the capsule of Cryptococcus neoformans.. Infect Immun.

[51] Diamond RD, May JE, Kane MA, Frank MM, Bennett JE (1974). The role of the classical and alternate complement pathways in host defenses against Cryptococcus neoformans infection.. J Immunol.

[52] Griffin FM (1981). Roles of macrophage Fc and C3b receptors in phagocytosis of immunologically coated Cryptococcus neoformans.. Proc Natl Acad Sci U S A.

[53] Janbon G, Doering TL, Heitman J, Kozel TR, Kwon-Chung KJ, Perfect JR, Casadevall A (2011). Biosynthesis and genetics of the Cryptococcal capsule.. Cryptococcus: From human pathogen to model yeast.

[54] Bose I, Reese AJ, Ory JJ, Janbon G, Doering TL (2003). A yeast under cover: the capsule of Cryptococcus neoformans.. Eukaryot Cell.

[55] Kozel TR, Gotschlich EC (1982). The capsule of cryptococcus neoformans passively inhibits phagocytosis of the yeast by macrophages.. J Immunol.

[56] Kozel TR, Pfrommer GS, Guerlain AS, Highison BA, Highison GJ (1988). Role of the capsule in phagocytosis of Cryptococcus neoformans.. Rev Infect Dis.

[57] Zhang JH, Chung TD, Oldenburg KR (1999). A Simple Statistical Parameter for Use in Evaluation and Validation of High Throughput Screening Assays.. J Biomol Screen.

[58] Zhang XD (2007). A new method with flexible and balanced control of false negatives and false positives for hit selection in RNA interference high-throughput screening assays.. J Biomol Screen.

[59] Zhang XD, Ferrer M, Espeseth AS, Marine SD, Stec EM (2007). The use of strictly standardized mean difference for hit selection in primary RNA interference high-throughput screening experiments.. J Biomol Screen.

[60] Birmingham A, Selfors LM, Forster T, Wrobel D, Kennedy CJ (2009). Statistical methods for analysis of high-throughput RNA interference screens.. Nat Methods.

[61] Kozel TR, Follette JL (1981). Opsonization of encapsulated Cryptococcus neoformans by specific anticapsular antibody.. Infect Immun.

[62] Pierini LM, Doering TL (2001). Spatial and temporal sequence of capsule construction in Cryptococcus neoformans.. Mol Microbiol.

[63] Daigneault M, Preston JA, Marriott HM, Whyte MK, Dockrell DH (2010). The identification of markers of macrophage differentiation in PMA-stimulated THP-1 cells and monocyte-derived macrophages.. PLoS One.

